# Effect of Location, Clone, and Measurement Season on the Propagation Velocity of Poplar Trees Using the Akaike Information Criterion for Arrival Time Determination

**DOI:** 10.3390/ma12030356

**Published:** 2019-01-24

**Authors:** Francisco J. Rescalvo, María A. Ripoll, Elisabet Suarez, Antolino Gallego

**Affiliations:** 1 Building Engineering School, University of Granada, Campus Fuentenueva s/n, 18071 Granada, Spain; rescalvo@ugr.es (F.J.R.); antolino@ugr.es (A.G.); 2 Andalusian Institute of Agricultural and Fisheries Research and Training (IFAPA), Camino del Purchil, 18004 Granada, Spain; mariaa.ripoll@juntadeandalucia.es

**Keywords:** wood, poplar, elastic waves, signal analysis, propagation velocity, entropy

## Abstract

The purchase price of any forest plantation depends on the quality of its raw wood, and specifically, variables such as density, orientation of the fibers, bending strength, and bending MoE (Modulus of Elasticity). The elastic waves propagation velocity has become one of the most popular parameters to evaluate the wood in standing trees. This study had two objectives: (1) Show how this velocity is clearly affected by the clone, the location of the crop, and the measurement season of poplar crops; and (2) apply the Akaike information criterion to determinate the arrival time of the waves, on the basis of the entropy of the signals recorded by the piezoelectric sensors placed on the trunk of the tree.

## 1. Introduction

Wood is a renewable construction material with a high potential for sustainability. In addition, due to numerous national and international guidelines for Energy Efficiency in Buildings, there is a strong interest in the use of wood in construction [1,2,3,4]. In this context, on the recommendation of FAO, plantations of fast-growing trees for fiber procurement, such as poplar, are becoming very important, since they also provide relevant ecosystem services (carbon sequestration, fight against climate change, soil conservation, and water cycle regulation) [5].

For the use of wood in construction, either in the sawn form or as part of engineered wood products (EWP), it is necessary to develop protocols and rigorous tools, for its characterization and categorization. Recent studies [6] indicate that if progress is made in the correct characterization and selection of wood from poplar crops, the engineering products derived from them may become specialized products, engineered for greater performance, thus, gaining profitability.

The purchase price of a forest plantation is directly related to the quality of its wood, which depends on variables such as density, orientation of the fibers, stiffness, mechanical strength, etc. [7]. Traditionally, density has been the parameter used to assess the quality of wood. However, for many applications, especially in the building sector, bending strength and bending MoE (Modulus of Elasticity) are key properties. The early and non-destructive determination of these properties has an important commercial and industrial interest, as, in this way, it will be possible to make a more efficient selection of logs, according to the final needs, and will provide a better economic value for the crop. Although it is very well-known from the physical point of view, [8] numerous studies have corroborated that these mechanical variables are directly related to the propagation velocity of artificially generated elastic waves in trees and logs [9,10,11,12,13,14,15,16,17].

In particular, there are three phases in which the propagation velocity of wood material can be assessed. Phase 1: On standing trees; Phase 2: On logs, once the tree has been harvested; and Phase 3: On planks or beams.

In Phase 1, the determination of the propagation velocity of the acoustic waves is usually carried out via the time of flight (ToF method) of a wave generated, with an actuator (usually one impact with a hammer) [18]. During Phases 2 and 3, the propagation velocity can be also be measured by stationary acoustic waves generated in the cut logs or planks (resonance method), through a spectral analysis [15,19,20,21,22]. There is some research work that has compared the longitudinal propagation speed obtained in Phase 1 (tree), with that obtained in Phase 2 (trunk), and in Phase 3 (planks/specimens), demonstrating a clear linear correlation between them [13,15,22]. Traditionally, the ToF method uses the threshold technique to determine the arrival time of the signal [3,17,23]. Alternatively, other methods like noise-reduction-based methods [24], higher-order statistics-based methods [25], floating threshold, Akaike information criterion [26], etc. have been proposed in the literature, for arrival time determination of transient signals. 

In this context, the goals of this paper are:

(1) Show how the propagation velocity measured by the ToF method, in the standing trees (Phase 1) is clearly affected by the clone, the location of the crop, and the measurement season, in the case of poplar crops located in South and North Spain. The authors do not know research work that has applied the ToF methodology to standing trees of cultivated poplar crops.

(2) Apply the Akaike information criterion as an alternative method to the classical threshold method, based on the entropy of the signals recorded by the piezoelectric sensors. This method, which has become very robust to the presence of noise in a signal and is especially suitable in a situation in which a smooth arrival of the wave occurs, has widely been used in other industrial sectors [27,28,29].

## 2. Methods and Materials

**Figure 1:**
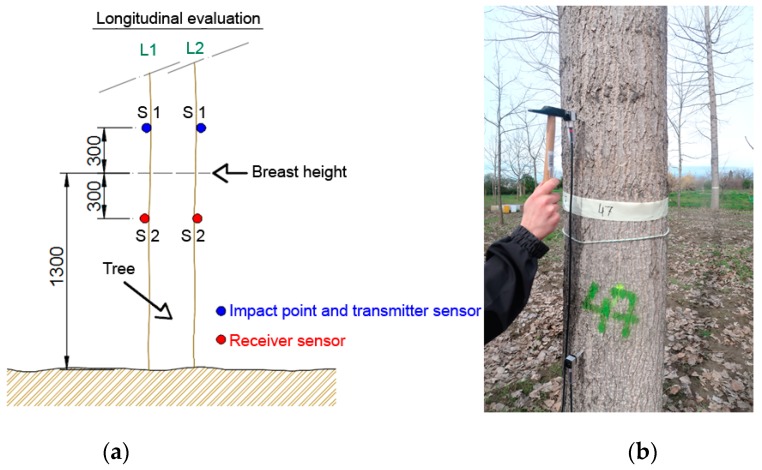
Time of Flight (ToF) inspection on the standing trees along directions L1 (N) and L2 (S). (**a**) Experimental setup; (**b**) Picture of a field test. Distances in mm.

In the traditional version of the ToF method, two piezoelectric transducers placed on the surface of the tree are used [13,15,20], although some authors have used three transducers [11,30,31,32]. The sensors are usually centered at the breast height (1300 mm) of the tree, as shown in Figure 1. The breast height (DBH) was measured by using a classic diameter caliper. Both transducers picked up the elastic waves generated by an impact with a hammer, carried out on the upper transducer (S1). If t_1_ and t_2_ are the arrival times of the signals received by both sensors, x_1_(t) and x_2_(t), the difference between both values Δt = t_1_ − t_2_, allows calculating the propagation velocity of the longitudinal elastic wave, in the outer wood of the tree [9,11,20], as C_T_ = d/Δt, d being the distance between the two transducers tips. Moreover, in order to ensure a good transmission of the wave, and thus, avoid the influence of the tree bark, the tips of the transducers were inserted at a particular distance. In addition, to enhance the generation and propagation of longitudinal waves, the tips of the transducers were inserted with an angle of 45°, with respect to the tree direction.

In this study, Fakoop’s SD-02 accelerometers, with a resonance of about 20 kHz, were used as transducers. The portable oscilloscope Picoscope^®^ with a bandwidth of 20 MHz and a sample rate of 80 MS/s was used to record the signals. Inspection was conducted in two directions along the tree, L1 and L2, oriented to the North and the South, respectively. Six tests were carried out along each direction, and the average value of the propagation velocity of the twelve measurements was calculated and used for the ulterior analysis. Following the indications of previous studies by other authors [17,33,34], the transducers were separated by 600 mm, and the tip was inserted 25 mm into the trunk. In order to evaluate the effect of the vegetative growth of the tree, the measurements were made during two different seasons of the year; in winter, with the sap stopped (March 2018), and in summer, with sap flowing (sap alive) (September 2018).

**Figure 2:**
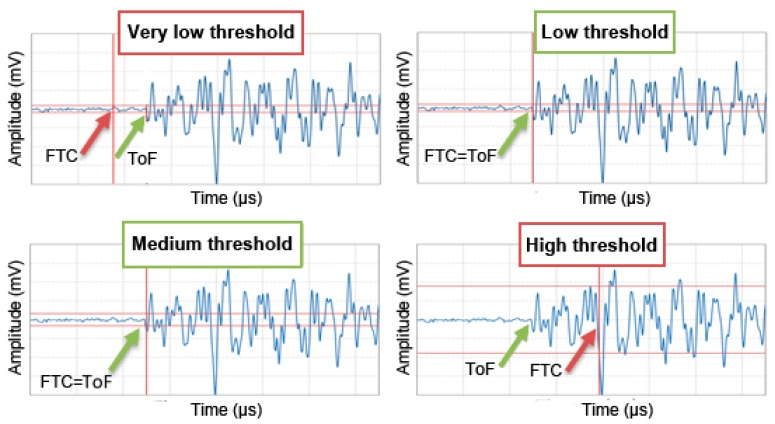
ToF and first threshold crossing (FTC) for different threshold values. Horizontal red line is the selected threshold. Vertical red line corresponds with the FTC; erroneous detection of the ToF (in red); and correct detection of the ToF (in green).

As it is obvious, the determination of the arrival time (ToF) of the signals x_1_ (t) and x_2_ (t) is a key aspect for the calculation of the propagation velocity. The traditional and easier method for determining the propagation velocity (threshold method) consists of establishing an amplitude value (threshold). Thus, the ToF, t_1_ or t_2_, is considered to be the first threshold crossing (FTC). However, this method is highly dependent on the chosen threshold. As shown in Figure 2, if the threshold is low, FTC will be lower than ToF, due to the presence of electrical noise. Otherwise, if the threshold is high, FTC will be greater than the ToF. Ideally, as it is deduced from Figure 2, FTC = ToF should be met.

As an alternative to the threshold method, the Akaike Information Criterion (AIC) has been applied in this paper. This method was developed in 1971 [35,36] and is especially suitable for transient signals [27,28,29]. The AIC can efficiently separate different events into a temporary signal, or otherwise detect the arrival time of the signal [27,28,29]. The method is based on the following argument—on the left of the arrival time, the signal is basically noise, which is characterized by high entropy. However, on the right of the arrival time, the noise has a low contribution, and therefore, the signal has a low entropy. That is, just at the ToF there is a strong entropy difference between the left and right portions of the signal.

In particular, the Akaike function—defined as the entropy difference signal between the left and right segments of the signal—is calculated as:

(1) AIC(k)=k log(variance(x(1:k))+(N−k−1)log(variance(x(k+1:N))

where N is the total number of data of the signal, and k is the order of each sample in the signal, which varies from 1 to N.

At the precise moment in which the dividing point of the two segments coincides with the arrival of the signal, the entropy difference will be maximum, and the Akaike function will reach its minimum value. In other words, the ToF is calculated as the time in which the AIC function reaches its minimum value.

**Figure 3:**
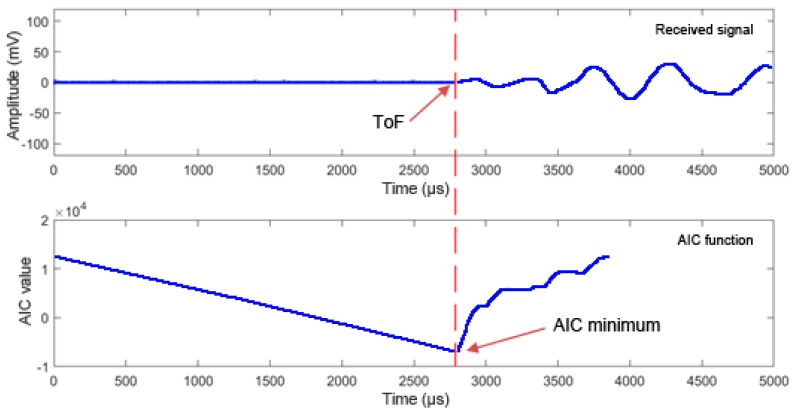
Example: ToF detection using the Akaike method.

**Figure 4:**
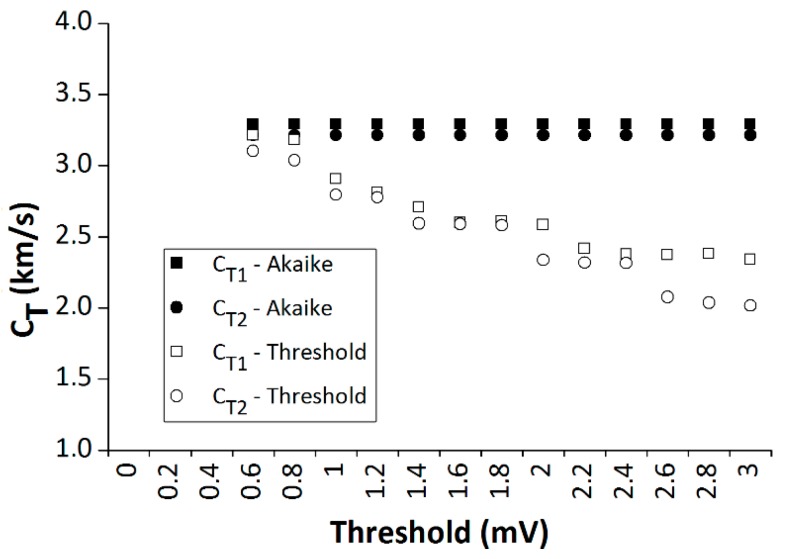
Propagation velocity as a function of the selected threshold. Comparison between the threshold and Akaike methods.

As an example, Figure 3 shows a signal received, x_2_ (t), by a transducer and the AIC function in a particular test. It can be observed that there is a perfect fit between the visually observed arrival time of the signal, the ToF, and the absolute minimum of the AIC function. Figure 4 shows a comparison of the propagation velocity, obtained along the two inspection directions, C_T1_ and C_T2_, between the threshold method and the Akaike method. Different threshold values are considered. It can be observed that the threshold method, unlike the Akaike method, produces velocities highly dependent of the chosen threshold.

This methodology (the ToF method with the determination of the arrival time using the Akaike function), was applied to obtain the propagation velocity of some trees of three different plantations located in Spain. Plantations 1 and 2 were located in Southern Spain, with higher average temperatures, fewer cloudy days, and greater availability of water, due to a very shallow water table that exists because of the presence of the Sierra Nevada Mountains. Plantation 3 was located in the North of Spain, with lower average temperatures, higher number of cloudy days, and less water availability. At the time of the measurements on the standing trees (ToF), plantations 1, 2, and 3 were 8, 5, and 13 years old, respectively. Table 1 summarizes the differences between the tree plantations. 

A more accurate description of the plantations is detailed below:

Plantation 1: This plantation was located in a private farm near Granada city, in Southern Spain. Average temperature was 11.4 °C in March and 21.6 °C in September. Climate was basically Mediterranean. This plantation belonged to the testing network of the Andalusian Institute of Agricultural and Fisheries Research and Training (IFAPA), Project PP.TRA.TRA2016.14. The trees were eight years old and the spacing between the trees was 5 × 5 m^2^ (the stocking in the stand was 400 sph-samples per hectare). The plantation was divided into two plots. In the first plot the I-214-clone poplar trees were alternated with hybrid walnut MJ209xRa trees (mix plot), which had a much slower growth than the poplars. The total number of poplars measured in this plot was 18. In the second plot there were only poplar trees of the I-214 clone (pure plot). Thirty-six trees were measured in this second plot. In both cases, the trees located at the edges of the plot were ignored. Clone I-214 (Populus x euroamericana (Dode) Guinier) is the most common variety in Europe and is typically used for the production of plywood panels.

Plantation 2: This plantation was also located near the city of Granada, in Southern Spain. The plantation also belonged to the testing networks of the IFAPA, Project PP.TRA.TRA2016.14. This plantation was 5 years old. It contained 180 poplar trees of four different clones, Unal (43 trees), Beaupre (45 trees), I-214 (50 trees), and Raspalje (42 trees); which were measured using the ToF method with the Akaike technique. The trees of the different clones were randomly distributed. The spacing between trees was also 5x5 m^2^ (the stocking in the stand was 400 sph). Similarly, the poplars placed at the edges of the plot were ignored for the analysis. 

**Figure 5:**
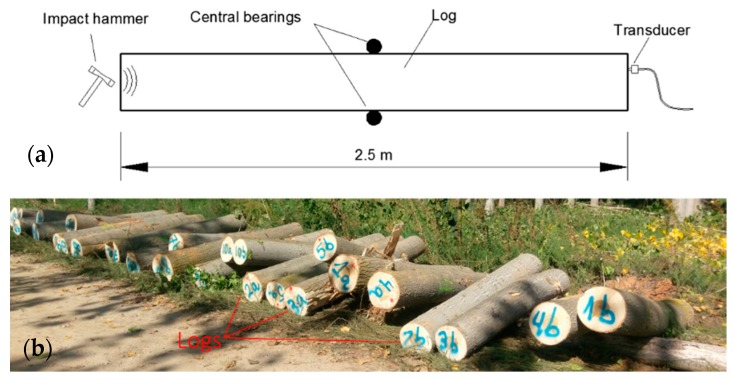
Resonance inspection on logs; the experimental setup (**a**); and image of the inspected logs (**b**).

Plantation 3: This plantation was located near the city of Yunquera de Henares (Guadalajara), in Northern Spain. The average temperatures in March and September was 8.6 °C and 18.7 °C, respectively. Spacing between the trees was 5.5 × 5.5 m^2^ (the stocking in the stand was 400 sph). This plantation was harvested in 2018. It was 13 years old. Before harvesting, an inspection of 15 poplar trees was carried out using the ToF method. After harvesting, a second inspection was made on 2.5 m logs (30 logs, two per tree), using the resonance method, as shown in Figure 5 [37].

## 3. Results and Discussion

### 3.1. Plantation 1: Influence of the Measurement Season and Type of Crop (Pure and Mix Crop)

**Figure 6:**
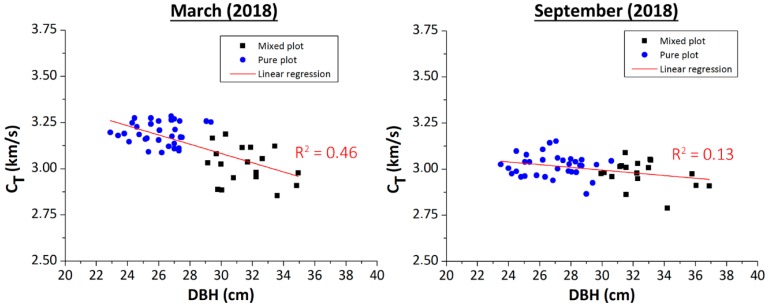
Propagation velocity versus DBH (diameter at breast height) for Plantation 1. Measurements during winter (March, 2018) and summer (September, 2018).

The average values of propagation velocity, obtained for each tree, using the Akaike method, versus the diameter of the tree measured at breast height (DBH), are shown in Figure 6. Likewise, a linear regression of C_T_ versus DBH is included. Table 2 summarizes the average values and the standard deviation of C_T_ and DBH for each type of plantation and season. They show that the season of the year in which the inspection of the standing trees was made, had a slight influence on the propagation velocity (the difference falls within the confidence intervals of the measures). This was due to the influence of the sap status. When the sap was stopped (winter season), velocity values were higher than when the sap was flowing (summer season). The greater presence of sap in summer, and therefore, greater humidity of the wood, caused a slight decrease of the propagation velocity [11,22].

It can be observed that the pure plantation trees reached smaller diameters, compared to the mixed plantation trees, in particular a 21.1% and 20.9%, for winter and summer measurements, respectively. This was because, in the mixed plot, the walnut trees helped the growth of poplar in its earliest years of life. During the first years of life, the walnuts experienced a very low growth, compared to the poplars, in such a way that the effective separation between the poplars (a species with growths much faster than that of the walnuts) was greater than 5 × 5 m^2^. This justified the highest DBH of the poplars in the mixed plot, with respect to the pure plot. In addition, the negative tendency of the propagation velocity, with respect to the DBH, demonstrated a greater stiffness of the wood of the thinner trees, compared to thicker trees, in good agreement with previous experimental studies on natural pine forests [10,11].

### 3.2. Plantation 2: Effect of the Clone

**Figure 7:**
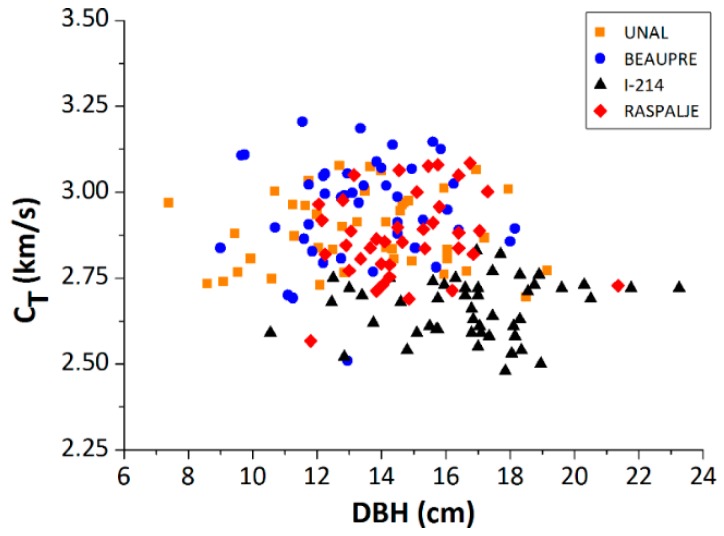
Propagation velocity versus DBH for the different clones of Plantation 2; Unal (orange square); Beaupre (blue circle); I-214 (black triangle); and Raspalje (red diamond).

Figure 7 shows the propagation velocity obtained for each clone tree versus DBH. The averages values and standard deviations are shown in Table 3. The measurements were made in summer, with sap alive (September 2018). In general, there was an influence of the clone on DBH and on the propagation velocity, in good agreement with previous analysis for other species [10,11,19,31,34]. Clone I-214, typically used in Europe for plywood panels, had a faster growth and provided greater tree diameters, but at the expense of a slower propagation velocity and, therefore, a lower stiffness of its wood. The clone I-214 provided a 21.5% higher DBH, compared to the rest of the clones studied, but with a reduction in velocity of 11%. Even so, this plantation was still young enough to be able to conclude significant differences between the different clones.

### 3.3. Plantation 3: Effect of the Crop Location

**Figure 8:**
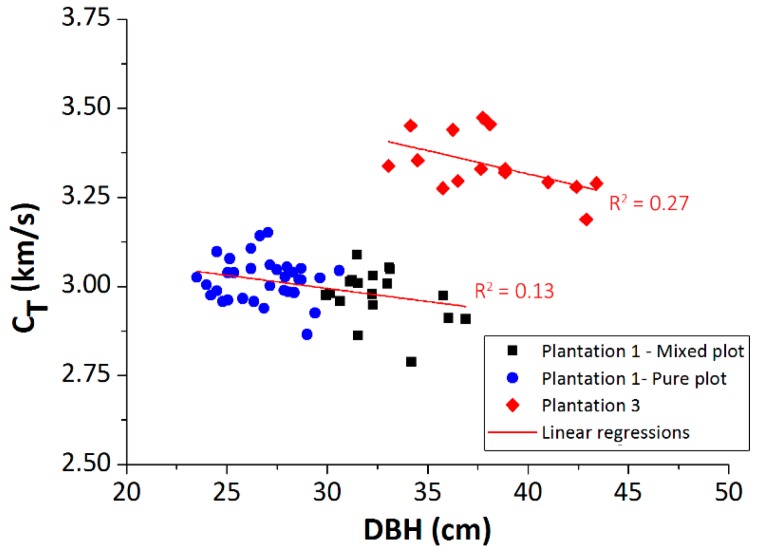
Propagation velocity versus DBH; Plantation 1—Walnut and Poplar sector (Southern Spain) (black square); Plantation 1—Poplar sector (blue circle); and Plantation 3 (Northern Spain) (red diamond).

Figure 8 shows the propagation velocity versus DBH for the trees of Plantation 3. Superimposed are the results of Plantation 1 (same clone, but different sand, and age). Table 4 summarizes the average DBH and propagation velocity values for each plantation. As it can be seen, there is a remarkable difference in the propagation velocity and DBH, between both plantations. In both cases, a similar negative relation between CT and DBH was observed. The DBH of Plantation 3 was 41.2% and 16.8% higher than the DBH of the pure and mixed plots of Plantation 1, respectively. Moreover, the propagation velocity of Plantation 3 was 11.5% higher than the mean velocity of Plantation 1 (the average velocity between the mixed and pure plots). These were basically due to three factors: (1) Plantation 3 was five years older than Plantation 1, which produced more adult wood and, therefore, with greater stiffness; (2) the stocking was higher in Plantation 1 than in Plantation 3, which entailed a greater competition among trees; and (3) Plantation 3 was located in a more extreme climate, with lower temperatures and lower water availability, which also produced a higher wood stiffness.

**Figure 9:**
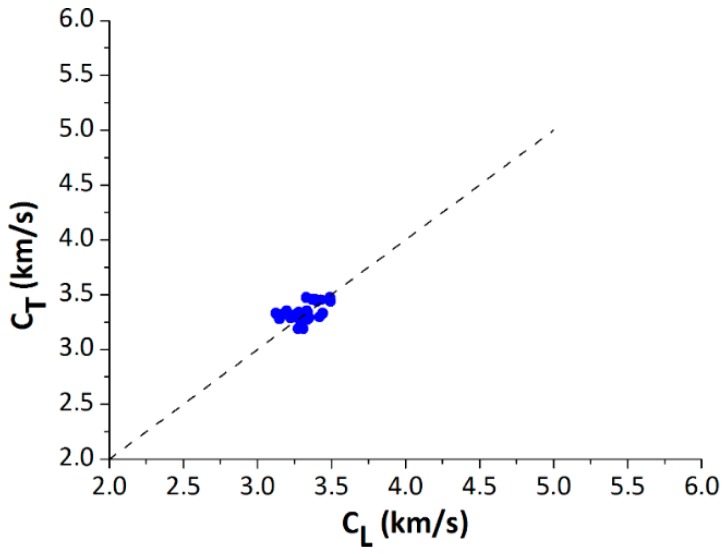
Propagation velocity of the standing trees (C_T_) versus the propagation velocity of the logs (C_L_), for Plantation 3.

Finally, as a validation of the technique, Figure 9 shows a comparison of the propagation velocity of standing trees, obtained by using the ToF method (C_T_) and the propagation velocity measured on the logs, by means of the resonance method (C_L_). It can be observed that the most of the points are grouped near the 45° line, i.e., CT ≅ CL, demonstrating a high homogeneity of the wood, due to the good management of the plantation (silvicultural practices). Average value of the C_T_ and the C_L_ was 3.34 and 3.32 km/s, respectively. It should be taken into account that the ToF method, and therefore, the C_T_, basically evaluates the stiffness of the outer wood, while the resonance method, C_L_, evaluates the wood of the entire log. Greater deviations between the C_T_ and C_L_ have been observed for other species, such as pine [20]. However, in the case of poplar, due to the low thickness of the bark, and the homogeneity of the wood, lower deviations between the inspection in trees and logs were shown by this study, i.e., C_T_ ≅ C_L_.

## 4. Conclusions

An analysis of the propagation velocity of standing trees obtained by means of the ToF (Time of Flight) method with the Akaike Information Criterion (AIC), to determine the arrival time of the waves, has been carried out on three different plantations of poplar trees of the I-214 clone, two located in Southern Spain (in the Granada province) and one in Northern Spain (in the Guadalajara province). 

It was found that the state of the sap (stopped or alive) had a slight influence on the propagation velocity. In winter (stopped sap) the velocity values were higher than in summer (sap alive), mainly due to the lower humidity of the wood. It was also shown that the presence of two species in the same plot, had an influence on the propagation velocity and the DBH. In particular, the walnut trees, had a slower growth in its first years, which allowed the poplar trees to have more space to grow, reaching higher values of DBH, compared to a pure poplar plantation. 

A negative relationship between the propagation velocity and DBH was verified, confirming previous results obtained on other softwood species. So, in general, trees with smaller DBH provide outer wood with greater stiffness.

A comparative study of different clones was carried out. Although the plantation was still young (5 years), the I-214 clone—the most used variety in Europe, for plywood—reached higher DBH values, compared to the rest of the clones, but provided lower values of propagation velocity. It was also verified that the climatological conditions and the watering amount of the plantation has a clear influence on the DBH and propagation velocity of the standing poplar trees.

However, it should be emphasized that although the same clone (I-214) was used in the three plantations, the age and plantation conditions were not similar. Hence, new and more extensive analyses and measurements must be carried out, in the future, to take this fact into account.

## Figures and Tables

**Table 1:** Characteristics of the different plantations.

Characteristics	Plantation 1	Plantation 2	Plantation 3
**Location (DMS)**	37°10′01.9″N 3°36′56.5″W	37°11′27.5″N 3°41′33.8″W	40°45′40.1″N 3°08′55.0″W
**Altitude (m)**	651	591	682
**Climate**	Transition between Mediterranean and Semi-arid	Transition between Mediterranean and Semi-arid	Continental Mediterranean
**Poplar clone**	I-214	Unal, Beaupre, I-214, and Raspalje	I-214
**Age (years)**	8	5	13
**Plantation density (m^2^)**	5 × 5	5 × 5	5.5 × 5.5
**Number of tested trees**	18 (mix plot) + 36 (pure plot)	43, 43, 50, and 39	15
**Measurement season**	March 2018 & September 2018	September 2018	September 2018

**Table 2:** Average values and standard deviation of DBH and propagation velocity for Plantation 1.

Parameters	March (2018)		September (2018)	
	Pure Plot	Mixed Plot	Pure Plot	Mixed Plot
**Average DBH (cm)**	26.1 ± 1.5	31.6 ± 1.8	27.0 ± 1.8	32.6 ± 2.0
**Average velocity (km/s)**	3.19 ± 0.06	3.02 ± 0.10	3.02 ± 0.06	2.97 ± 0.07

**Table 3:** Number of trees, average values, and standard deviation of DBH and propagation velocity for each poplar clone.

Parameters	Unal	Beaupre	I-214	Raspalje
**Number of trees**	43	45	50	42
**Average DBH (cm)**	13.4 ± 2.7	13.4 ± 2.1	16.8 ± 2.4	14.7 ± 1.9
**Average velocity (km/s)**	2.89 ± 0.11	2.95 ± 0.15	2.66 ± 0.08	2.86 ± 0.12

**Table 4:** Average values of DBH and propagation velocity for Plantations 1 (Southern Spain) and Plantation 3 (Northern Spain).

Parameters	Plantation 1		Plantation 3
	Pure Plot	Mixed Plot	
**Average DBH (cm)**	27.0 ± 1.8	32.6 ± 2.0	38.1 ± 3.2
**Average velocity (km/s)**	3.02 ± 0.06	2.97 ± 0.07	3.34 ± 0.08
